# The (Sialyl) Tn antigen: Contributions to immunosuppression in gastrointestinal cancers

**DOI:** 10.3389/fonc.2022.1093496

**Published:** 2023-01-06

**Authors:** Christabelle Rajesh, Prakash Radhakrishnan

**Affiliations:** Eppley Institute for Research in Cancer and Allied Diseases, University of Nebraska Medical Center, Omaha, NE, United States

**Keywords:** Tn antigen, STn antigen, glycosylation, immune cells, tumor microenvironment (TME), pancreas-adenocarcinoma, gastrointestinal tumour

## Abstract

Cellular signaling pathways are intricately regulated to maintain homeostasis. During cancer progression, these mechanisms are manipulated to become harmful. O-glycosylation, a crucial post-translational modification, is one such pathway that can lead to multiple isoforms of glycoproteins. The Tn (GalNAc-O-Ser/Thr) and Sialyl Tn (STn; Neu5Ac-GalNAc-O-Ser/Thr) antigens resulting from the incomplete synthesis of fully branched O-glycan chains on proteins contribute to disease progression in the pancreas and other gastrointestinal cancers. The tumor microenvironment (TME) is a major constituent of tumors and a key modulator of their behavior. Multiple cellular and secretory components of the TME dictate the development and metastasis of tumors. Immune cells like macrophages, natural killer (NK) cells, dendritic cells, B and T lymphocytes are a part of the tumor “immune” microenvironment (TIME). The expression of the Tn and STn antigens on tumors has been found to regulate the function of these immune cells and alter their normal antitumor cytotoxic role. This is possible through multiple cell intrinsic and extrinsic signaling pathways, elaborated in this review. Studying the interaction between Tn/STn antigens and the TIME of gastrointestinal cancers can help develop better and more robust therapies that can counteract immunosuppressive mechanisms to sensitize these tumors to anticancer therapies.

## 1 Introduction

The advancement of proteomics and its different tributaries, like phospho-proteomics, has facilitated the understanding of the structural and functional roles of various proteins in cancer cells. Protein function is influenced by many cellular signals and post-translational modifications (PTMs). Glycosylation is a ubiquitous and prominent PTM, with glycan structures found on secretory and membrane-bound proteins. The reactions generating glycans are catalyzed by enzymes called glycosyltransferases that reside in the endoplasmic reticulum, Golgi apparatus, and some extracellular spaces (1, 2). In humans, the two main glycosylation pathways are N-glycosylation, in which a monosaccharide is attached to the nitrogen atom of Asparagine on proteins, and O-glycosylation, wherein the linkage is *via* oxygen atoms on Serine or Threonine amino acid residues in proteins (3). Not surprisingly, the pathogenesis of cancer works hand in hand with important glycan alterations on a variety of proteins. For example, N-glycan modifications have been found to stabilize the PD-L1 immune checkpoint and decrease cytotoxic T-cell activity (4). O-glycosylation-based changes are very abundant in the evolution of cancer. One of the most abundantly expressed aberrant O-glycoforms is the Tn antigen and its derivative, the STn antigen (5, 6). These antigens arise from incomplete O-glycan synthesis, leading to a dearth of fully branched O-glycans that can be used to the advantage of tumor cells. The multitude of mechanisms that can lead to the presence of aberrantly glycosylated proteins and important events altered by these Tn and STn antigens to hijack tumor cell signaling in gastrointestinal cancers like pancreatic ductal adenocarcinoma (PDAC) and influence the infiltration of immune cells in the tumor niche are discussed in this review.

## 2 What is the Tn/STn antigen?

**Figure 1:**
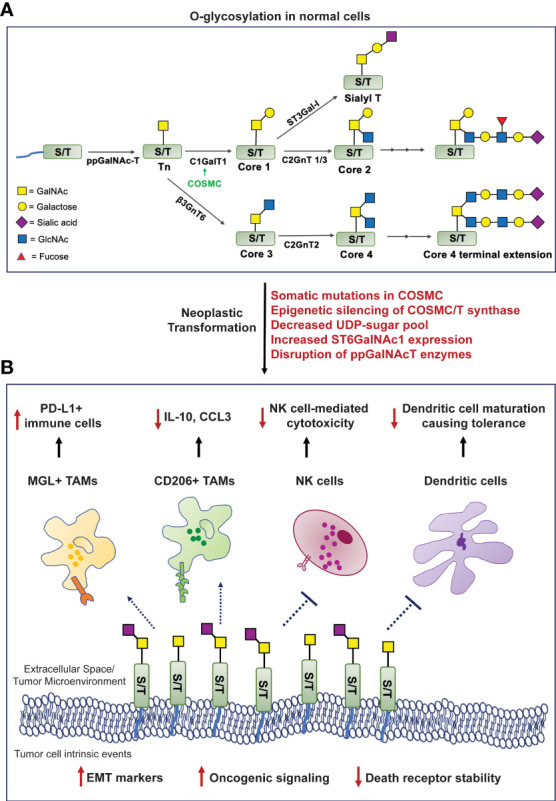
Formation of truncated O-glycans in tumors modulates cell-intrinsic behavior and interactions with the tumor immune microenvironment. **(A)**. O-glycosylation in human cells involves highly regulated sequential reactions catalyzed by glycosyltransferases. **(B)**. Mechanisms mediating loss of O-glycan chain extension (mentioned in red) facilitate neoplastic transformation, increase oncogenic signaling cascades, the epithelial-to-mesenchymal transition process, and compromise death receptor stability to evade apoptosis. The Tn/STn antigens interact with macrophage-galactose lectin (MGL) and CD206 (mannose receptor), expressing tumor-associated macrophages (TAMs); natural killer (NK) cells; and monocytic dendritic cells - preventing their maturation, leading to an overall immunosuppressed microenvironment. [ppGalNAc-T, polypeptide-GalNActransferase; C1GalT1, core1 β3-galactosyltransferase; COSMC, Core1-Specific-Molecular-Chaperone; C2GnT, core2 β1,6 N-acetylglucosaminyltransferase; β3GnT6, β-1,3-N-Acetylglucosaminyltransferase; ST6GalNAc-1, α2,6 sialyltransferase; ST3Gal-1, α2,3 sialyltransferase; PDAC, pancreatic ductal adenocarcinoma].

The process of O-glycosylation is highly complex and regulated by a series of enzymatic reactions catalyzed by glycosyltransferases initiated in the Golgi apparatus. The most exposed amino acid residues on a folded protein in the Golgi are Serine (Ser) and Threonine (Thr). O-glycosylation begins with the addition of an N-acetylgalactosamine (GalNAc) residue using UDP-GalNAc by the enzyme polypeptidyl α-GalNAc transferase (ppGalNAcT) to a Ser/Thr residue to form a covalent bond at their hydroxyl group. This structure is called the Tn antigen (GalNAc-α1-O-Ser/Thr), also referred to as the Thomsen-nouveau antigen (7). Further addition of a galactose (Gal) residue to Tn forms the T antigen, also known as the Core 1 structure (Galβ1-3-GalNAc-α1-O-Ser/Thr) or the Thomsen-Friedenreich (TF) antigen. This is catalyzed by the enzyme T synthase (synonyms: core1 β3-galactosyltransferase, C1GALT1). T synthase is synthesized in the endoplasmic reticulum (ER), and its proper folding is ensured by a chaperone called COSMC (Core1-Specific-Molecular-Chaperone). In cellular conditions devoid of a functional T synthase, adding one sialic acid (N-acetylneuraminic acid or Neu5Ac or NANA) residue to the GalNAc generates the STn antigen, a reaction catalyzed by the sialyltransferase ST6GalNAc-I (8). On the other hand, elongation of the T antigen or Core 1 structure forms the Core 2 O-glycan branching. Additionally, the Tn antigen can be modified to create the Core 3 and Core 4 structures using a different set of glycosyltransferases, all of which lead to complex O-glycosylation-based branching in normal cells (**Figure 1A**).

## 3 Molecular mechanisms leading to generation of the Tn/STn antigens in cancer

The aberrant overexpression of the Tn/STn antigens is observed in many diseases and malignancies. Some of the mechanisms that can mediate this process are as follows: (i) Somatic mutations in COSMC can prevent proper folding of T synthase (C1GALT1) and render it incapable of extending the Tn structure (9); (ii) Epigenetic silencing of either COSMC or T synthase transcription as identified in the “Tn syndrome” in hematopoietic cells and pancreatic cancer (10); (iii) Aberrant expression or subcellular localization of the ppGalNAcT enzymes (11, 12); (iv) Loss of availability of the necessary UDP-sugars to facilitate glycan chain extension post Tn/STn antigen formation (13); (v) Elevated expression of the ST6GalNAc-I enzyme that can outcompete T synthase and promote the terminal generation of the STn antigen, in a way that inhibits further addition of any sugar by the glycosyltransferases (14) (**Figure 1A**).

## 4 Effect of the Tn/STn antigen on cell signaling in cancer

Cancer models like cell lines have been used to study various mechanisms that govern the pathogenesis of tumors. In one such expedition, researchers studying the molecular basis of breast cancer discovered the overexpression of the Tn/STn antigens in this malignancy for the first time (15). The Tn/STn antigens have since been established as members of the tumor-associated carbohydrate antigen (TACA) group of cancer biomarkers due to their overwhelming presence in various solid tumors (16). Radhakrishnan et al. reported for the first time that pancreatic cancer cells possess a hypermethylated promoter for the gene encoding COSMC in about 40% of PDAC patients studied in their cohort. This leads to the downregulation of T synthase and heightened Tn/STn antigen expression. These cancer cells exhibit a highly invasive and migratory phenotype due to altered cell-cell adhesion structures that disrupt tissue homeostasis and instigate tumorigenic processes (17). The Tn/STn antigen’s tumor-promoting role was further validated in robust cell line and orthotopic models of pancreatic cancer. COSMC knockout led to tumor spread as it enhanced the epithelial-to-mesenchymal transition (EMT), a critical hallmark of cancer, and increased stemness markers, including CD133 and CD44 in PDAC cells (18). In another study, Dong et al. knocked out the gene encoding C1GALT1 in colorectal cancer cells, which prevented the expression of the T-synthase enzyme, leading to the expression of the Tn/STn antigen. Markers like N-cadherin, Snail, and Slug were all elevated in the C1GALT1 knockout cells demonstrating the higher EMT potential of these Tn/STn expressing cells as compared to the counterparts expressing the C1GALT1 (**Figure 1B**) (18). Additionally, the presence of the Tn/STn antigens on the glycoprotein MUC16 increased aggressiveness in PDAC. Tn/STn-MUC16 bound to the α4β1 integrin complex more efficiently than its fully glycosylated counterpart, and augmented integrin-linked kinase (ILK) and focal-adhesion kinase (FAK) mediated cell signaling that increased cell survival and migration (19). A brief description of mucin-associated truncated O-glycans in the context of immune evasion is described in a further section.

The death receptors (DR) 4 and 5 interact with ligands TRAIL/Apo2L and stimulate programmed cell death. There exist crucial conserved O-glycosylation sites on these death receptors, and mutations in these sites lead to disrupted O-glycan presence on the receptor ectodomains, making them ineffective in inducing apoptosis (20). Jiang et al. showed that a similar mechanism was prevalent in cancer cells deficient in COSMC, expressing the Tn/STn antigen. Tn/STn expression attenuated TRAIL-induced apoptosis in cancer cells - more specifically, by impairing homo-oligomerization and structural stability of the DR4/5 receptors. Treating these cells with the TRAIL/Apo2L ligands rendered the cancer cells insensitive to cell death. Thus, the Tn/STn antigens can facilitate the escape of tumor cells from apoptosis (21). Expression of COSMC in such Tn-positive cells *via* transfection facilitated the production of a functional T-synthase, which enabled the extension of Core 1 and Core 2 O-glycan structures. Treating the cancer cells now containing functionally active COSMC/T-synthase with TRAIL ligand-mediated their apoptosis and decreased neoplastic transformation (**Figure 1B**) (21, 22).

## 5 Tn/STn antigen-induced immune modulation

### 5.1 Immune cells affected by the Tn/STn antigens

O-glycosylation and its involvement in immune modulation is a field that has seen immense scrutiny in the last three decades. In 1992, Ogata et al. demonstrated the impact of the mucin associated STn antigen on natural killer (NK) cells in colon cancer. This mechanism was illustrated using ovine submaxillary mucin (OSM), which is known to be aberrantly O-glycosylated *via* the expression of the STn antigen. OSM significantly inhibited NK cell activity when combined with ammonium-based treatment, while removing the sialyl groups from this mucin decreased such NK cell inhibition (**Figure 1B**) (23). Other synonymous signaling pathways elicited by the Tn/STn antigens, which result in immunosuppression and facilitate aggressive tumor progression, are highlighted below.

Mucins comprise a group of glycoproteins, some of which – including MUC1 and MUC16, are highly upregulated in gastrointestinal cancers. Escape from immune cell-mediated killing is one of the potential mechanisms of “protection” mucins offer to the tumor (24–27). Mucins and their O-glycans are involved in macrophage-based tumor infiltration. The mucin CA125 (MUC16) is highly overexpressed in multiple solid tumors – and had been initially extensively studied in ovarian cancer (28). Tumor-associated macrophages (TAMs) isolated from ovarian cancer patients express the C-type lectin mannose receptor (MR), also known as CD206. This MR on the TAMs interacts with the STn-positive regions and MUC16 on tumor cells, which leads to the production of the anti-inflammatory cytokine IL-10 and a decrease in the T cell attracting inflammatory chemokine CCL3, facilitating immune suppression (**Figure 1B**) (29, 30). CD206 is also heavily expressed by TAMs in PDAC and is found to mediate immunosuppression, possibly through such Tn interaction, and facilitate disease progression (31). An abnormally high expression of the Tn antigen has also been reported in patients with high-grade glioblastoma. As with most other cancers, the expression of the Tn antigen is specific to these cancer cells and does not appear in normal brain cell counterparts. The TAMs in Tn-positive glioblastomas interact with the Tn antigen *via* the macrophage-galactose lectin (MGL), which is also a C-type lectin receptor. This further instigates the infiltration of PD-L1-positive immunosuppressive macrophages (**Figure 1B**) (32).

Studying the glycosylation landscape of tumors has been deemed important in validating immunotherapies for cancer (33). A recent study investigating the glyco-code of PDAC and its role in immune modulation found that ligands (namely, the Tn antigen on PDAC cells) for the MGL receptor are highly expressed on both epithelial and mesenchymal cells of the tumor in PDAC patients. Simultaneous activation of the MGL receptor (by the Tn antigen) and the DC-SIGN receptor (by fucosylated antigens) on tumor-associated macrophages (TAMs) triggered the generation of IL-10 with a concomitant decrease in IL-6, leading to a “tolerogenic tumor microenvironment”, incapable of mounting a potent anti-PDAC response (**Figure 1B**) (33, 34). The MGL receptor is also expressed on dendritic cells (35). MUC1 is highly overexpressed in PDAC and is correlated with poor prognosis (26). Napoletano et al. showed that MUC1-associated Tn antigen (Tn-MUC1) binds the MGL receptor on immature monocyte-derived dendritic cells (imDCs). Once bound, the Tn-MUC1 glycopeptide is internalized and processed through the HLA class I and II pathways. This proves that the Tn-MUC1 antigen is a potent immunogen that can be presented to the immune system *via* dendritic cells. Interestingly, the non-glycosylated MUC1 protein was incapable of binding to the MGL receptor, which further highlights the importance of tumor cell surface glycan modifications (36). The main component of the antigenic Tn-MUC1 that facilitates such imDC-expressing MGL-based antigen presentation is the terminal GalNAc residue. A specific YENF motif in the cytoplasmic domain of the human MGL receptor present on dendritic cells is essential for binding to the terminal GalNAc and promoting the endocytosis of the Tn-MUC1 glycopeptide (37). Hence, through this mechanism, the Tn antigen can increase the infiltration of T cells *via* antigen presentation through dendritic cells and result in antitumor cytotoxicity. In another study, bladder cancer cells expressing the STn antigen were found to have higher tumorigenic properties than STn-negative cells and bound imDCs more strongly. However, such STn-mediated interactions prevented dendritic cell maturation leading to a lack of antigen presentation and subsequently induced tolerance, evidenced by reduced antitumor Th_1_ cells and increased regulatory T cell populations, demonstrating cancer-/context-dependent mechanisms elicited by these antigens (38).

Recently, Cornelissen et al. demonstrated how the Tn antigen modifies the immune landscape in colorectal cancer (CRC) model using the MC38 cell line with the C1GALT1 gene knocked out. The subsequent high Tn antigen-expressing tumors were more aggressive, and expression of the Tn antigen correlated with a decrease in CD8^+^ cytotoxic T cell infiltration and an increase in the myeloid-derived suppressor cells (MDSCs), the latter of which is accruing more interest with researchers investigating immunosuppressive cells in the tumor niche (39). Another study in a CRC model demonstrated a positive correlation between the expression of the Tn antigen and a mismatch repair deficient state (MMRd) in CRC cells, along with a “cold” or immunosuppressed microenvironment (40).

### 5.2 The interaction between STn antigen and sialic acid binding lectins (Siglecs) modulates immune cell functions

As discussed above, the generation of the STn antigen is catalyzed by the enzyme ST6GalNAc-I to form a sugar chain terminating in a sialyl group. Overexpression of this enzyme has been observed in a plethora of cancers, leading to hyper-sialylated O-glycan structures (41). This has aroused a lot of research to evaluate the role of Siglecs (Sialic-acid binding immunoglobulin superfamily lectins) in these cancers. Siglecs are membrane-bound proteins that contain carbohydrate-binding domains. Humans have a total of 14 distinct functionally dynamic Siglecs. Of these, Siglec-1, Siglec-2, Siglec-4, and Siglec-14 are highly conserved. The other receptors belonging to this family include Siglecs – 3, -5, -6, -7, -8, -9, -10, -11, 15, and -16. Of the 14 known Siglec receptors, 9 contain ITIM (immunoreceptor tyrosine-containing inhibitory motif) or ITIM-like domains in their cytoplasmic signaling tails and can mediate immunosuppressive mechanisms (42). Interestingly, these four Siglecs contain ITIM and ITIM-like domains in their signaling components. The text below highlights the function of Siglec family receptors in modulating immune suppression in gastrointestinal cancers.

The STn antigen is recognized by Siglec-6, expressed in the placenta and the uterine endometrium, and is thought to play a role in labor (43). In mast cells isolated from colon cancer models, Siglec-6 was one of the immune inhibitory receptors to be upregulated. Upon binding colon cancer cell-associated ligands, Siglec-6 mediated attenuation of mast cell degranulation/cytotoxicity. Such a mechanism was further elevated in a hypoxic environment, frequently observed in solid tumors like colon cancer (44).

**Figure 2:**
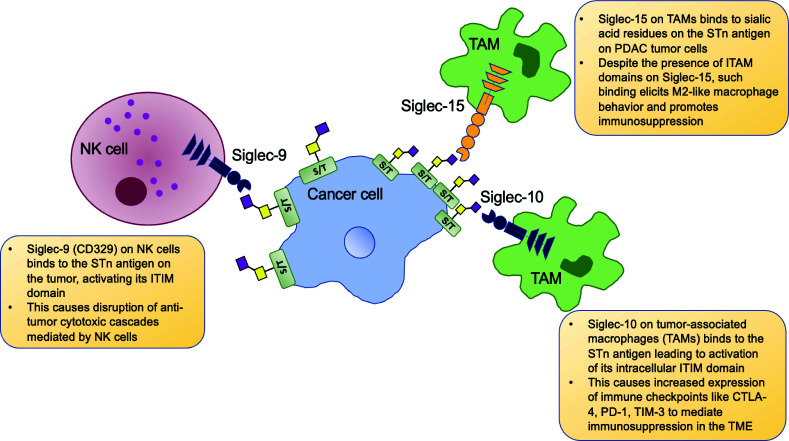
Tumor cell STn-specific interactions with immune cells. Cancer cells overexpress the STn antigen and interact with Siglec receptors on immune cells and activate their intracellular ITIMs (immunoreceptor tyrosine-based inhibitory motifs). Siglec-9 on NK cells (and monocytes, not shown here) bind with STn antigens present on PDAC and ovarian tumor cells and cause disruption of NK cell-mediated tumor cytotoxicity. Siglec-10 expressing tumor associated macrophages (TAMs) bind the STn antigen on hepatocellular carcinoma cells, leading to upregulation of immune checkpoints and overall immune suppression in the tumor microenvironment (TME). Siglec-15 expressing TAMs in the PDAC TME bind STn antigen on the tumor and such Siglec-15: STn binding overrides the immunoreceptor tyrosine-based activation motif (ITAM) based signaling *via* Siglec-15 and instead mediates M2-like macrophage polarization, bringing about heavy immunosuppression.

Along with ovarian cancer, the mucin MUC16 is highly overexpressed in more than 60% of PDAC tumors and significantly worsens disease progression in this highly lethal malignancy (27). Siglec-9, another ITIM and ITIM-like domain-containing receptor, is expressed in monocytes, NK cells, and B cells. Monocytic and NK cell Siglec-9 was found to strongly interact with STn antigen containing MUC16 to mediate immunosuppression in ovarian cancer and exacerbate prognosis (**Figure 2**) (45). Interestingly, alpha2,3 sialic acid expressing MUC1 was also found to bind Siglec-9 expressed on myeloid cells and mediate TAM phenotypes and cancer immunosuppression (46). Given the high expression of both these mucins in PDAC and other solid tumors, this highlights the important role of truncated O-glycan-bearing mucins in mediating immune evasion in cancers. Another study showed that glycan sialylation was high in PDAC tumors. Interaction between Siglecs-7/9 on monocytes with the PDAC tumor (specifically alpha2,3 and alpha2,6 sialic acid, i.e., STn antigen) drove their conversion to TAMs that ultimately promoted tumor progression (47).

While studying the TME of hepatocellular carcinoma (HCC), Xiao et al. found a high proportion of Siglec-10 expressing macrophages in patient specimens with poor survival outcomes. These Siglec-10^high^ TAM bearing tumors showed increased anti-inflammatory immunosuppressive markers, including PD-1, CTLA-4, and TIM-3. A mAb targeting Siglec-10 was able to reverse such immunosuppression in HCC by downregulating the immune checkpoints and increasing IFN-y and IL-2 secretion to ultimately augment CD8+ cytotoxic T cells (**Figure 2**) (48).

Siglec-15 is an evolutionarily conserved receptor that recognizes the STn antigen (49). A recent study demonstrated that TAMs in PDAC express Siglec-15 and can bind the alpha2,6 sialic acid STn antigen (along with alpha2,3 sialic acid) expressing domains on tumor cells. Such interactions promote Siglec-15 mediated Syk signaling and immunosuppressive M2 polarization of the macrophages. Furthermore, sialidase treatment and subsequent loss of terminal alpha2,3 and alpha2,6 sialic acids rendered the macrophages less susceptible to being polarized to M2-like tumor-promoting TAMs (50). Siglec-15 has been shown to be a potent blocker of CD8+ T cells, and hence, such siglec-mediated immunosuppression is a crucial regulator of tumor progression (**Figure 2**) (51).

## 6 Antibodies targeting the (S)Tn antigen

Antibodies are crucial components of our response against pathogens. They bring about cytotoxic effects by various mechanisms: (i) Antibody-dependent cellular cytotoxicity (ADCC); (ii) Complement-mediated cytotoxicity (CDC); (iii) Blocking receptors on the surface of cancer cells that lead to dampening of tumor-promoting signaling. In 1981, the first monoclonal antibody (mAb) against the Tn antigen (B72.3) was generated in mice challenged with membrane fractions isolated from human breast cancer samples (52). One of the epitopes recognized by B72.3 is a mucin-like glycoprotein TAG-72, which is routinely used to assess the presence of the Tn antigen in research. As TAG-72 is an antigen that binds Tn/STn targeting antibody, this protein was purified and then administered to mice, which led to the generation of another mAb called CC49, which also reacts with the Tn and STn antigens (53). The CC49 mAb has been tagged with a radiolabel Lutetium-177 and subsequently tested in clinical trials in combination with interferon-α and paclitaxel for the treatment of ovarian cancer (54). Many other mAbs that react with the Tn and STn antigens have since been developed for cancer screening and therapy. One such antibody, TKH2, targeted the STn antigen and was used to coat polymeric nanoparticles loaded with cisplatin. Targeted delivery of this compound increased gemcitabine sensitivity to STn-high PDAC tumors (55). NC318 is another mAb in phase I/II clinical trials (NCT03665285) that blocks Siglec-15 and is being employed as a therapeutic strategy for metastatic solid cancers, including CRC, cholangiocarcinoma and more. Preclinical studies with NC318 demonstrated its ability to alleviate tumor burden and immunosuppression, partly by re-establishing T cell cytotoxicity (56). Gatipotuzumab is another mAb used in phase I/II clinical trials for solid tumors (NCT03360734, NCT01222624) that binds to STn-bearing MUC1 and prevents its interactions with siglec-9, to instigate ADCC against the tumor cells (57, 58).

## 7 Conclusions and perspectives

The role of the Tn and STn antigens in the pathogenesis of gastrointestinal malignancies discussed in this review is manifold. These antigens are expressed on multiple proteins, especially mucins, through various molecular mechanisms that affect the activity of the T synthase, COSMC, and/or ST6GalNAc-I enzymes. Mostly, the expression of these aberrant O-glycoforms leads to tumor-promoting mechanisms. These mechanisms are initiated through a wide range of cell signaling cascades that tumor cells use for their own benefit. In recent literature, the focus has been directed on the role of Tn/STn antigens in affecting components of the tumor microenvironment. The role of the Tn/STn antigens in modulating the behavior of immune cells has ramifications that need to be combated to boost anti-cancer treatment strategies in Tn-high tumors. These antigens predominantly mediate multiple immunosuppressive schemes that facilitate the development of what we now know as an “immune cold” tumor. Fortunately, many targeted therapies are being developed to attenuate such pro-tumor immune escape strategies. This area of glycoproteomic research has seen relatively slower growth because of factors like the low availability of suitable model systems until the last two decades to monitor the immune fraction and issues met with the development of potent molecules like antibodies to detect the Tn/STn antigens. The challenges associated with these therapies have been acknowledged, and further research to dissect the exact mechanisms involved in disease resistance is being avidly conducted.

## Author contributions

The manuscript was written and designed by CR and PR. Both authors reviewed and approved the final manuscript.
